# Identification and characterization of *Loa loa* antigens responsible for cross-reactivity with rapid diagnostic tests for lymphatic filariasis

**DOI:** 10.1371/journal.pntd.0006963

**Published:** 2018-11-16

**Authors:** Marla I. Hertz, Hugues Nana-Djeunga, Joseph Kamgno, Abdel Jelil Njouendou, Valerine Chawa Chunda, Samuel Wanji, Amy Rush, Peter U. Fischer, Gary J. Weil, Philip J. Budge

**Affiliations:** 1 Infectious Diseases Division, Department of Medicine, Washington University School of Medicine, St. Louis, Missouri, United States of America; 2 Centre for Research on Filariasis and other Tropical Diseases, Yaoundé, Cameroon; 3 Faculty of Medicine and Biomedical Sciences, University of Yaoundé 1, Yaoundé, Cameroon; 4 Parasites and Vector Biology Research Unit (PAVBRU), Department of Microbiology and Parasitology, University of Buea, Buea, Cameroon; 5 Research Foundation for Tropical Diseases and the Environment (REFOTDE), Buea, Cameroon; University of Zurich, SWITZERLAND

## Abstract

The Global Program to Eliminate Lymphatic Filariasis (LF) relies on rapid diagnostic tests (RDTs) to determine where annual mass drug administration for LF is required and when it can be stopped. These tests detect a *Wuchereria bancrofti* glycoprotein in the blood of infected persons via a carbohydrate moiety recognized by the monoclonal antibodies AD12 and DH6.5. Loiasis cross-reactivity with LF RDTs has recently been recognized as a serious obstacle to LF elimination in loiasis-endemic areas. To better understand the nature of this cross-reactivity, we used the DH6.5 antibody to immunoaffinity purify *Loa loa* antigens from the sera of individuals with a positive RDT due to loiasis. Immunoblot analysis revealed many circulating AD12/DH6.5-reactive antigens, and proteomic analysis identified multiple *L*. *loa* proteins in LF RDT-positive loiasis sera. These included both secreted and somatic proteins, suggesting that they may be released by dying *L*. *loa* adult worms and/or microfilariae. Unlike the single high molecular weight *W*. *bancrofti* circulating filarial antigen that is reliably present in the blood of persons with bancroftian filariasis, reactive *L*. *loa* antigens appeared to be only transiently present in the blood of a subset of persons with loiasis. These key differences between the circulating antigens of *W*. *bancrofti* and *L*. *loa* can be used to differentiate positive results generated by both species and may lead to improved diagnostic tests for LF and loiasis.

## Author summary

Lymphatic filariasis is a disfiguring parasitic infection tens of millions of people in more than 70 countries. The global effort to eliminate LF transmission via mass drug administration (MDA) relies on rapid diagnostic tests (RDTs) to identify infected individuals and map afflicted areas. This effort is complicated in loiasis-endemic nations of central Africa for two reasons. First, persons with heavy *L*. *loa* infections may suffer severe adverse events, including death, following treatment with MDA medications. Second, it is now clear that RDT testing for LF can be unreliable in areas with loiasis, since many *L*. *loa-*infected individuals, especially those with heavy infections, test positive by LF RDT in the absence of infection with *W*. *bancrofti* (the causative agent of LF in Africa). We report here the identity and characteristics of multiple *L*. *loa* antigens found in RDT-positive sera that bind to antibodies used in LF RDTs. Understanding the differences between these cross-reactive antigens and the circulating filarial antigen of *W*. *bancrofti* may lead to development of improved diagnostic tests for LF and loiasis to facilitate elimination of filarial infections in Sub-Saharan Africa.

## Introduction

Lymphatic filariasis (LF) is a disabling and disfiguring disease caused by mosquito-borne, filarial (threadlike) parasitic worms. The Global Program to Eliminate LF (GPELF) reduced the at-risk population for LF from 1.2 billion to 789 million (a 46% reduction) between 2000 and 2012 by providing repeated, annual rounds of anti-filarial medications by mass drug administration (MDA). However, LF elimination in Africa lags behind other endemic regions with only a 25% reduction [1]. This is due in part to slow rollout of MDA in regions of central Africa co-endemic with *Loa loa*, because drugs used for MDA can cause severe adverse effects in people with loiasis [2].

The GPELF strategy relies on point of care rapid diagnostic tests (RDTs) to map regions endemic for LF and to determine when regions have successfully eliminated the disease. Two RDTs have been used to detect a circulating antigen of *Wuchereria bancrofti*, the filarial species that causes LF in Africa. These are the Binax NOW Filariasis immunochromatographic card test (ICT), and the Alere Filariasis Test Strip (FTS). The FTS is more stable, more sensitive, and less expensive than the ICT [3], but both are lateral flow assays that work as follows. Whole blood is applied to a sample pad containing colloidal gold-conjugated polyclonal antifilarial antibodies. The sample pad retains blood cells, while capillary action pulls the serum across the test strip and over a test line containing an immobilized IgM class monoclonal antibody called AD12. The AD12 antibody binds a carbohydrate epitope that is abundantly present on a high molecular weight (200–250 kDa) *W*. *bancrofti* circulating filarial antigen (CFA) [4, 5]. AD12 traps *W*. *bancrofti* CFA bound to the colloidal gold-labeled polyclonal antibodies to form a pink line that indicates a positive test result.

The molecular structure and saccharide composition of the carbohydrate epitope recognized by the AD12 antibody is unknown. This carbohydrate moiety, which we refer to as the AD12 epitope, appears to be specific to nematodes and is not phosphorylcholine [5]. A second IgM monoclonal antibody developed in the Weil laboratory, DH6.5, also recognizes the AD12 epitope [5], and we use these two antibodies interchangeably. Like the AD12 carbohydrate epitope, the exact identity of the high molecular weight *W*. *bancrofti* CFA remains unknown. It is clear, however, that it contains multiple AD12 epitopes per molecule, since capture of the molecule by DH6.5 in an ELISA format does not prevent AD12 / DH6.5 from binding to the non-bound surface of the molecule [6]. While other nematodes have antigens that contain the AD12 epitope [5], the ICT and FTS tests have been considered functionally specific for *W*. *bancrofti* infection, since until recently, such antigens had not been found circulating in the blood of persons with other infections.

It has now become clear, however, that some persons with loiasis, especially those with high *L*. *loa* microfilaria (Mf) counts, have positive LF RDT results [7–9]. This complicates mapping and monitoring for LF elimination programs in central Africa. Therefore, the purpose of this study was to identify filarial antigens in the blood of persons infected with *L*. *loa* that could cause false-positive LF RDT results. This information may lead to the development of improved diagnostic tests for both infections.

## Materials and methods

### Ethics statement

This research involved the testing of adult human sera. Sample collection was approved by the institutional review board of Washington University in St. Louis based on protocols 201512112 for Akonolinga/Awae and 201512016 for the East Region collections in Cameroon. The studies were also approved by the Cameroon National Ethics Committee and Ministry of Public Health. Written informed consent was obtained from all participants.The *L. loa* microfilariae harvested from baboons kindly provided by S. Wanji were approved by the Ministry of Scientific Research and Innovation of Cameroon (Research permit #028/MINRESI/B00/C00/C10/C12). Animal experiments were conducted within the guidelines stated by the animal care and use committee at the National Institutes of Health (USA).

### Sample collection

**Fig 1:**
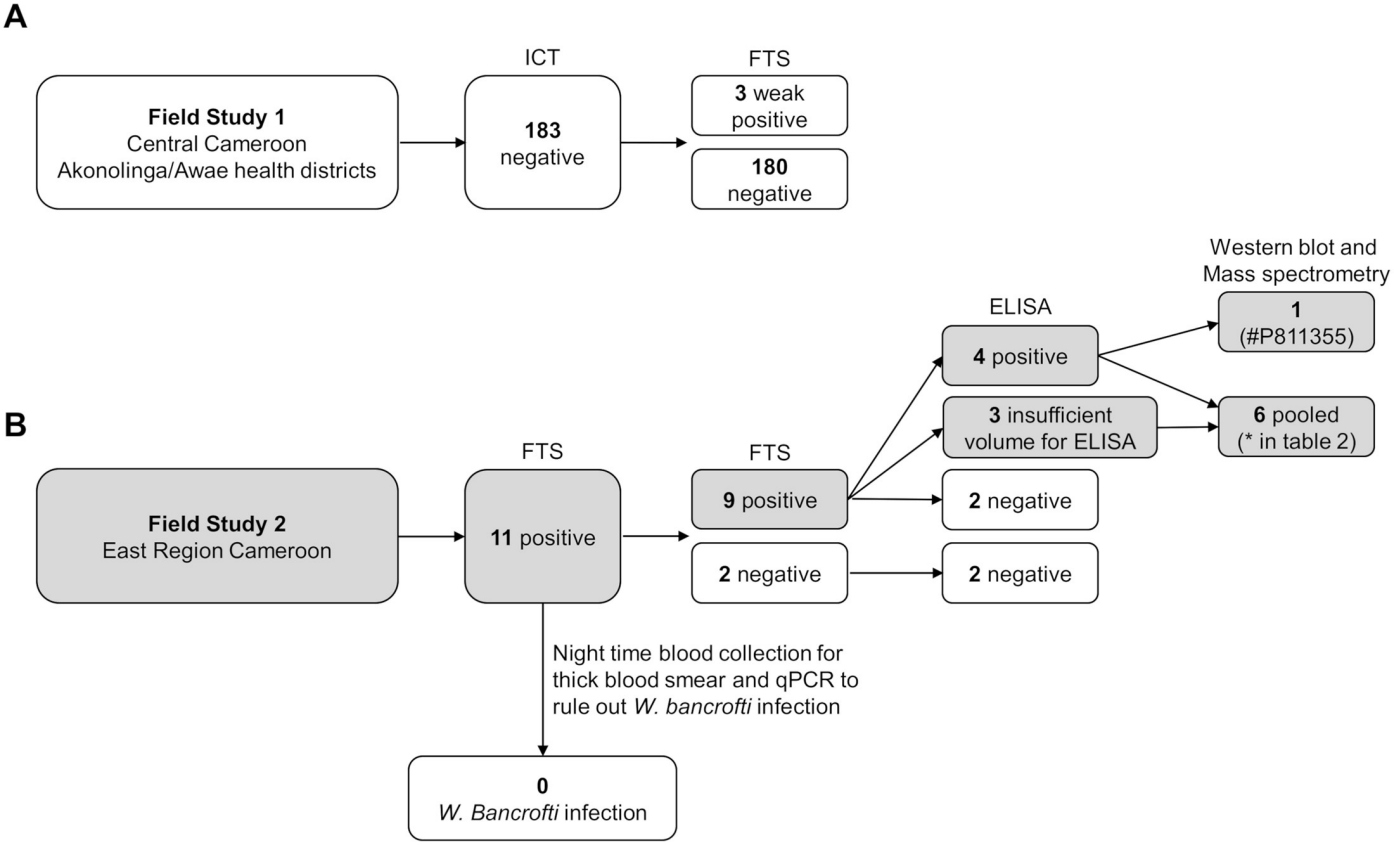
Field studies and samples tested. **(A)** Central Cameroon field study. Because the three FTS positive samples were very weakly positive by FTS and negative by ICT, we chose not to examine them further. **(B)** East Region study. Participants who were ICT-positive on initial screening were visited the following week for venous blood collection. Nine venous blood samples were FTS-positive. Two of these tested negative for filarial antigen by ELISA; the other seven were tested further by ELISA and/or western blot as indicated. Gray shading indicates the origin of the samples used in the proteomic analysis.

We collected plasma and serum samples in two field studies (Fig 1). The first was conducted in the Akonolinga and Awae health districts (Central Region of Cameroon) in February 2016, for the specific purpose of obtaining serum samples from persons with loiasis that were positive in LF RDTs. The second study, conducted in the East Region of Cameroon in December 2016, was part of an effort to re-map areas of Cameroon for the presence of *W*. *bancrofti* and *L*. *loa*. Both study areas are endemic for *L*. *loa* and *Mansonella perstans*, but most likely not for *W*. *bancrofti* (Wanji and co-workers, pers. commun.). Neither area had received community-based ivermectin treatment or MDA for LF prior to these studies.

The goal of the Akonolinga/Awae study was to obtain serum samples containing cross-reactive *L*. *loa* antigens from at least ten persons with positive ICT tests due to loiasis. We planned a sample size of 200 participants, based on the assumption that about 10% of persons with *L*. *loa* microfilaremia would have a reactive test [7]. We specifically recruited adults with known loiasis in prior studies, but also invited adults with unknown infection status living in the same villages to participate in order to generate a biobank of loiasis positive and negative samples. Potential participants gathered at a central location in each village (usually the health center) and were informed of the purpose of the study. Consenting participants underwent day (10 AM to 2 PM) and night (10 PM to 2 AM) blood collections. Fresh fingerprick capillary blood was used for ICT testing (100 μL) and for preparation of thick blood smears (70 μL). Dried blood spots were collected for subsequent PCR testing (90 μL, applied to HemaSpot cartridges, Spot On Sciences, Inc., Austin, TX). Venous blood (~10 mL) was collected in K2 ethylenediaminetetraacetic acid (EDTA) vacutainer tubes (BD Biosciences, Franklin Lakes, NJ), stored overnight at 4°C, and then separated by centrifugation. Following separation, plasma was stored at 4°C for up to 3 days prior to transport to Yaoundé, then stored at -80°C. Dried blood spots and aliquots of plasma samples were shipped on dry ice to Washington University in St. Louis for further analysis. After finding that none of the participants in the first study were ICT-positive (see Results), we obtained sera from eleven FTS-positive participants of an initially unrelated study in the East Region of Cameroon. The purpose of the East Region study was to re-map the prevalence of LF and loiasis in Cameroon, and the primary results of that study will be published separately. The re-mapping study was conducted in the Lomie, Doume and Nguelemendouka health districts. Following a brief interview, consented individuals underwent serological and parasitological testing. Daytime (between 10 AM and 4 PM) capillary blood was tested for CFA by FTS (70 μL) and for Mf by thick calibrated thick blood film smears (TBS, 50 μL). Daytime venous blood (4 mL) was collected from FTS-positive individuals in vacutainer tubes without anticoagulant, and sera were separated using a centrifuge and stored in cryovials at -20°C for up to 7 days in the district facilities prior to transport to Buea, where they were stored at -80°C. Night TBS were prepared between 10 PM and 2 AM from participants that tested positive by FTS. Night blood dried onto filter paper was used for detection of parasite DNA by qPCR, as reported previously [7, 10]. Sera from the re-mapping study were shipped on dry ice to Washington University in St. Louis for further analysis. Loiasis-negative banked sera (collected outside of Africa) from persons with microfilaremic *W*. *bancrofti* infections [11, 12] were used as positive controls for CFA tests.

### Detection of Mf in blood samples

Thick blood smears were stained with Giemsa, and the number of *L*. *loa* and *M*. *perstans* Mf were counted as previously described [13]. *M*. *perstans* were distinguished from *L*. *loa* based on morphology and size. Each slide was read by two experienced microscopists.

### Quantitative PCR

qPCR was performed at Washington University as previously described [7]. Briefly, three blades of HemaSpot finger prick blood were used for DNA extraction by QIAamp DNA mini kits (Qiagen, Valencia, CA) Real-time PCR using Taqman Multiplex master mix (Applied Biosystems, Foster City, CA) was performed with an ABI Quant 6 instrument under standard conditions using primer and probe sequences as previously described [10, 14].

### *W*. *bancrofti* RDTs

The ICT (BinaxNow Filariasis) and Filariasis Test Strip (FTS) (both purchased from Alere, Scarborough, ME) diagnostic tests for circulating filarial antigen were performed according to the manufacturer’s instructions and read 10 minutes after applying the sample to the sample pad [3]. Positive results were recorded with a semi-quantitative scale in which: 0 = no test (T) line visible (negative), 1 = the T line was visible but weaker than the control (C) line, 2 = the T line was equal to the C line, and 3 = the T line was stronger than the C line.

### In vitro culture of *L*. *loa*

*L*. *loa* Mf were isolated and purified from venous blood collected from experimentally infected baboons [15]. Immature adult stage (L5) *L*. *loa* were isolated from Rag2^-/-^/IL2γc^-/-^ BALB/c mice 90 days after subcutaneous inoculation of 100 L3 stage worms. Pools of parasites (40,000 Mf and 5 L5) were incubated in 400 μl serum-free RPMI medium supplemented with 2mM L-glutamine, 2 g/L Na_2_HCO_3_ with 10% (v/v) penicillin-streptomycin-neomycin mixture in a 37°C/ 5% CO_2_ incubator. Culture supernatants (ES) applied directly to FTS after incubation for 32 hours (Mf) or 24 hours (L5) to detect the presence of reactive antigens.

### Filarial antigen ELISA

Detection of circulating filarial antigen by sandwich ELISA was performed as previously described [6]. This assay uses two IgM monoclonal antibodies, AD12 and DH6.5, both of which recognize the AD12 carbohydrate epitope. Reagents for ELISA, immunoprecipitation and immunoblot experiments were purchased from Sigma-Aldrich (St. Louis, MO), unless otherwise noted.

### Competition assay for antibody to the AD12 epitope

*Brugia malayi* excretory secretory products (Bm-ES) were prepared by incubating adult female *B*. *malayi* worms (obtained from the Filariasis Research Reagent Resource Center, Athens, GA) in serum free RPMI 1640 media, supplemented with L-glutamine and penicillin/streptomycin (Gibco) in a 37°C/ 5% CO_2_ incubator. The spent media was collected every other day while worms were viable (approximately 2 weeks) and Bm-ES was concentrated by centrifugal filtration using an Amicon Ultra 3000 MWCO (Millipore) filter. Polyvinyl, U-bottom microtiter plates (Thermo Electron Corp., Milford, MA) were coated overnight at 37°C with 100 μl/well of Bm-ES product diluted to 1.25 μg/mL in 0.6M carbonate buffer, pH 9.6. After coating, wells were washed with phosphate buffered saline (PBS) containing 0.05% Tween 20 (PBS-T) then blocked with 200 μL 5% fetal calf serum in PBS-T. Wells were then incubated with 50 μL of a 1:5 dilution of test sera or unlabeled DH6.5 antibody (7μg/mL) for one hour at 37°C, washed with PBS-T to remove unbound antibody, and incubated with 100 μL peroxidase-conjugated AD12 antibody for one hour at 37°C. Wells were washed three times with PBS-T, and 100 μL O-phenylenediamine dihydrochloride (OPD) substrate added, developed for ten minutes, stopped by adding 30 μL of 4M sulfuric acid, and absorbance read at 492 nm.

### Immunoprecipitation

Five mg of antibody DH6.5 was directly conjugated to 1mL packed Affigel 10 beads (Bio-Rad, Hercules, CA) according to the manufacturer’s protocol. Fifty microliters of conjugated beads were mixed with 100–750 μl of each human serum sample (depending on the amount of sample available), then incubated at 4°C with rocking overnight. The beads were then washed four times with cold PBS, re-suspended in 1X NuPAGE LDS sample buffer (Invitrogen), and heated to 95°C for five minutes to release bound antigens. For *L*. *loa* ES products, one mL of *in vitro* culture supernatant was mixed with 50 μL DH6.5-conjugated beads and rocked at 4°C overnight. The beads were washed four times with cold PBS, four times with a high salt, detergent buffer (MIB-T buffer: 5mM HEPES, 1M NaCl, 1mM EDTA, 1mM EGTA, 10mM NaF, 2.4mM NaVO_4_, 0.5% Triton X-100 + protease inhibitor cocktail), then twice in cold PBS to remove the detergent. Antigens were released by heating in 1X NuPAGE LDS sample buffer as written above.

### Polyacrylamide gel electrophoresis and western blot analysis

Proteins were resolved by SDS-PAGE using a 4–12% bis-tris NuPAGE gel (Invitrogen) and transferred to nitrocellulose membranes (Cell Signaling Technology, Danvers, MA). Membranes were incubated 1 hour at room temperature with PBS-T containing 5% nonfat, dried milk to block nonspecific binding and then incubated with a 1:1000 solution of peroxidase-conjugated AD12 antibody in blocking buffer for one hour at room temperature. Blots were washed three times in PBS-T, incubated with Clarity Western ECL substrate (Bio-Rad, Hercules, CA), and bound antibody detected by chemiluminescence using a Bio-Rad ChemiDoc instrument and Image Lab 5.2.1 software. The *L*. *loa* soluble antigen (Loa Ag) used as a positive control in the immunoblots was prepared by grinding adult *L*. *loa* worms in extraction buffer (10mM Tris pH 8.3, 2% sodium deoxycholate, 1mM PMSF, 1mM EDTA, 1mM EGTA, 25ug/mL TLCK protease inhibitor, 15ug/mL TPCK protease inhibitor). The adult worms were a gift from Dr. Vida Dennis, Tulane University.

### Liquid chromatography-tandem mass spectrometry (LC-MS/MS)

Immunoprecipitation samples were prepared for LC-MS/MS using the filter-aided sample preparation method [16]. Briefly, samples were reduced with DTT and buffer-exchanged into 8M urea/Tris, alkylated with 40mM iodoacetamide, then buffer-exchanged in several steps to 0.05M NH_4_HCO_3_ using a Microcon 30K filter concentrator (Millipore, Billerica, MA). Samples were treated with PNGase F for 1–3 hours to remove N-linked carbohydrates. The samples were then resuspended in urea/tris, reduced with TCEP, incubated with iodoacetamide to block reactive thiols, and buffer-exchanged back into 0.05M NH_4_HCO_3_. Trypsin, chymotrypsin, or both were added and samples were incubated overnight at 37°C, after which the digested peptides were centrifuged through the 30K filter and desalted using a Glygen C4 tip (Glygen, Columbia, MD). LC-ESI/MS/MS analysis was performed using a Q-Exactive Plus Hybrid Quadrupole-Orbitrap Plus mass spectrometer (ThermoFisher Scientific) coupled to an EASY-nanoLC 1000 system (ThermoFisher Scientific). The samples were loaded (2.5 μL) onto a 75 μm × 50 cm Acclaim PepMap 100 RP column (ThermoFisher Scientific). The peptides were eluted at a flow rate of 300 nL/min with an acetonitrile gradient in aqueous formic acid (0.1%) as mobile phase A. Peptide elution occurred in the following sequence: 0–4% buffer B (Acetonitrile containing 0.1% formic acid) for 1 min, 4–12% over 63 minutes, 12–22% B over 56 minutes, 22–30% B over 20 minutes, 30–70% B over 6 minutes, followed by increase in B to 95% B over 1 min and an isocratic wash at 95% B for 12 min. Full-scan mass spectra were acquired by the Orbitrap mass analyzer in the mass-to-charge ratio (*m/z*) of 375 to 1500 and with a mass resolving power set to 70,000. Twelve data-dependent high-energy collisional dissociations (HCD) were performed with a mass resolving power set to 35,000, a fixed first *m/z* 100, an isolation width of 1.2 *m/z*, and the normalized collision energy (NCE) setting of 27. The maximum injection time was 120 ms for parent-ion analysis and 120 ms for product-ion analysis. Target ions already selected for MS/MS were dynamically excluded for 30 sec. An automatic gain control (AGC) target value of 3 x 10^6^ ions was used for full MS scans and 5 x 10^5^ ions for MS/MS scans. Peptide ions with charge states of one or greater than seven were excluded from MS/MS acquisition. The resulting MS spectra were converted to Mascot generic format (MGF) using Proteome Discoverer v2.1.0.81. MGF files were submitted for peptide identification against target databases available for *L*. *loa* (Bioprojects PRJNA246086 [17] and PRJNA60051 [18] downloaded from WormBase ParaSite (parasite.wormbase.org) on Feb 27, 2017), contaminant databases from ENSEMBL for Human (Homo_sapiens.GRCh37.72 ENHU) and Mouse (Mus_musculus.GRCm38.72 ENMOU), and the cRAP database version (2012.01.01) for common contaminating peptides (http://www.thegpm.org/crap/). The search engine used was PEAKS Studio 8.0 build 20160908. The following parameters were used during database search: Oxidation of methionine was allowed as variable modification; carbamidomethylation of cysteine as a fixed modification; maximum of 3 missed cleavages; trypsin and chymotrypsin as the proteolytic enzymes depending on the sample; MS1 error tolerance of 20.0 ppm and MS2 error tolerance of 0.02Da. Qualifying peptides had a less than 1% false discovery rate and were absent from the combined control databases with human, mouse and common contaminating peptides.

### Bioinformatic analysis

Blast2Go software was used to assign Gene Ontology (GO) terms to the combined immunoprecipitation (IP) dataset and to the whole genome of *Loa* (PRJNA246086) to build a reference database [19]. NetOGlyc 4.0 was used to predict O-glycosylation sites in the hits from the mass spec analysis. These results were compared with the O-glycosylation prediction of 500 randomly selected proteins from the genome to determine enrichment of O-glycoproteins [20]. The same approach used to predict N-glycosylation sites with NetNGlyc 1.0 [21]. SignalP 4.1 software was used to predict classical N-terminus secretion signals and SecretomeP 2.0 was used to predict non-classical secretion signals [22, 23]. Reciprocal BLAST searches with the *B*. *malayi* genome (BioProject PRJNA10729, parasite.wormbase.org) were used to identify *B*. *malayi* homologs of the *L*. *loa* antigens identified in the proteomics screen. The resulting dataset was used for a meta-analysis of *B*. *malayi* proteomics studies as a proxy to characterize the *L*. *loa* proteins.

## Results

### Loss of ICT cross-reactivity in residents of Akonolinga/Awae

Prior filariasis surveys in the Akonolinga and Awae health districts in Central Cameroon area had shown 1–5% ICT positivity with a *W*. *bancrofti* microfilaremia prevalence of 0.23%. This result suggested that most, if not all, of the ICT positives were due to *L*. *loa* cross reactivity [24]. In February 2016, we collected daytime and nighttime blood samples from 183 persons. This included 89 persons who had tested positive for loiasis in prior studies, and 18 who were previously ICT positive (tested in either 2013 or 2015; S1 Table). One hundred eleven participants (59%) had *L*. *loa* microfilaremia in blood collected during the day, and 71 (41%) of these also had nocturnal *L*. *loa* microfilaremia. No participant had *W*. *bancrofti* microfilaremia by microscopy and only one sample, which was FTS negative, tested positive for *W*. *bancrofti* DNA by qPCR. Surprisingly, none of the participants were ICT positive, including the 18 who had been ICT positive in prior studies, even though *L*. *loa* Mf counts in these participants had not decreased (Table 1). We also tested plasma from each participant by FTS; and only three samples were weakly positive (1+ test line).

**Table 1:** Central Cameroon field study *L*. *loa* and *W*. *bancrofti* test results by prior ICT status.

Prior ICT *	ICT+	FTS+	*L*. *loa* PCR+	*Wb* PCR+	Median *L*. *loa* Mf/mL (IQR)	
					Prior results	Current results
ICT+ (N = 18)	0	1	15 (83%)	0	11,870 (5,240–32,540)	17,540 (12,120–36,620)
ICT- (N = 30)	0	0	13 (43%)	0	2,720 (840–6,900)	2,000 (340–4,360)
No prior (N = 135)	0	2	38 (28%)	1	N/A	40 (0–3,880)

*Prior ICT tests were in either 2013 (N = 24) or 2015 (N = 24)

### Loss of reactivity is not due to masking antibody

**Fig 2:**
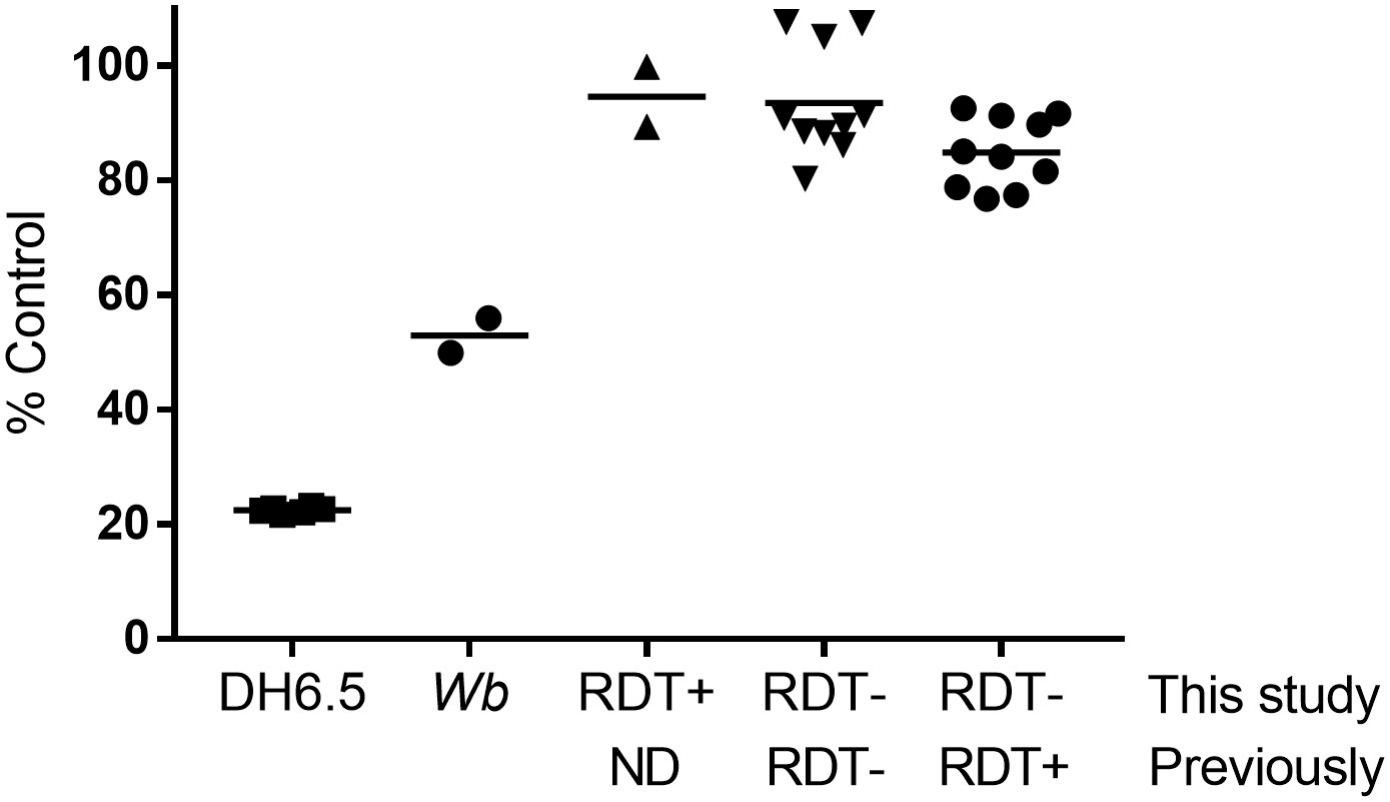
Loiasis patient sera lack antibodies specific to the AD12 carbohydrate epitope. Serum samples were tested for the ability to block AD12 binding to *B*. *malayi* antigens that contain the AD12 epitope. The horizontal line denotes the mean value for each group.

It has been observed that the absence of circulating filarial antigen in some *W*. *bancrofti-*infected individuals is related to the host humoral response to the AD12 epitope [6]. To investigate whether the lack of *W*. *bancrofti* RDT cross-reactivity in the previously ICT-positive participants was due to development of antibodies against the AD12 epitope in these persons, we tested a subset of samples for anti-AD12 epitope antibody by competition ELISA. None of the samples reduced binding of the AD12 antibody to AD12 epitopes by more than 20%, regardless of the current or prior ICT/FTS status of the individual (Fig 2). This suggests that the lack of RDT-positivity in the previously positive samples is not due to the presence of AD12 epitope-masking antibodies.

### East Region samples

Given the lack of cross-reactive antigenemia in the Akonolinga/Awae participants, we also tested banked sera from eleven ICT-positive participants in an integrated LF re-mapping study conducted in the East Region of Cameroon in 2016. These participants all had *L*. *loa* microfilaremia (Table 2) but no *W*. *bancrofti* Mf by nocturnal thick blood smear and no detectable *W*. *bancrofti* DNA by qPCR. All 11 participants also had *M*. *perstans* microfilaremia. Nine of the eleven sera were FTS positive, and four had detectable AD12 epitope-containing antigen by CFA ELISA (Table 2 and Fig 1).

**Table 2:** Summary of Mf burden and antigenemia in ICT/FTS cross-reactive *L*. *loa* infected individuals from East Region Cameroon mapping study.

Sample ID#	*L*. *loa* (Mf/mL)	FTS score	CFA (ng/mL)	*M*. *perstans* (Mf/mL)
P811473	13600	0	0	1200
P811092	41650	0	0	900
P811331*	9750	1	5.8	1150
P811493*	7700	1	ND	1950
P811461*	21450	1	81.4	8650
P811408*	44350	1	ND	6650
P811377*	74350	1	86.3	1800
P811161	50100	1	0	550
P811264	16800	2	0	950
P811134*	59500	2	ND	1000
P811355	82950	2	>400	900

*Serum pooled in further analyses

ND = insufficient sample volume to conduct CFA ELISA

### Western blot analysis of cross-reactive sera

**Fig 3:**
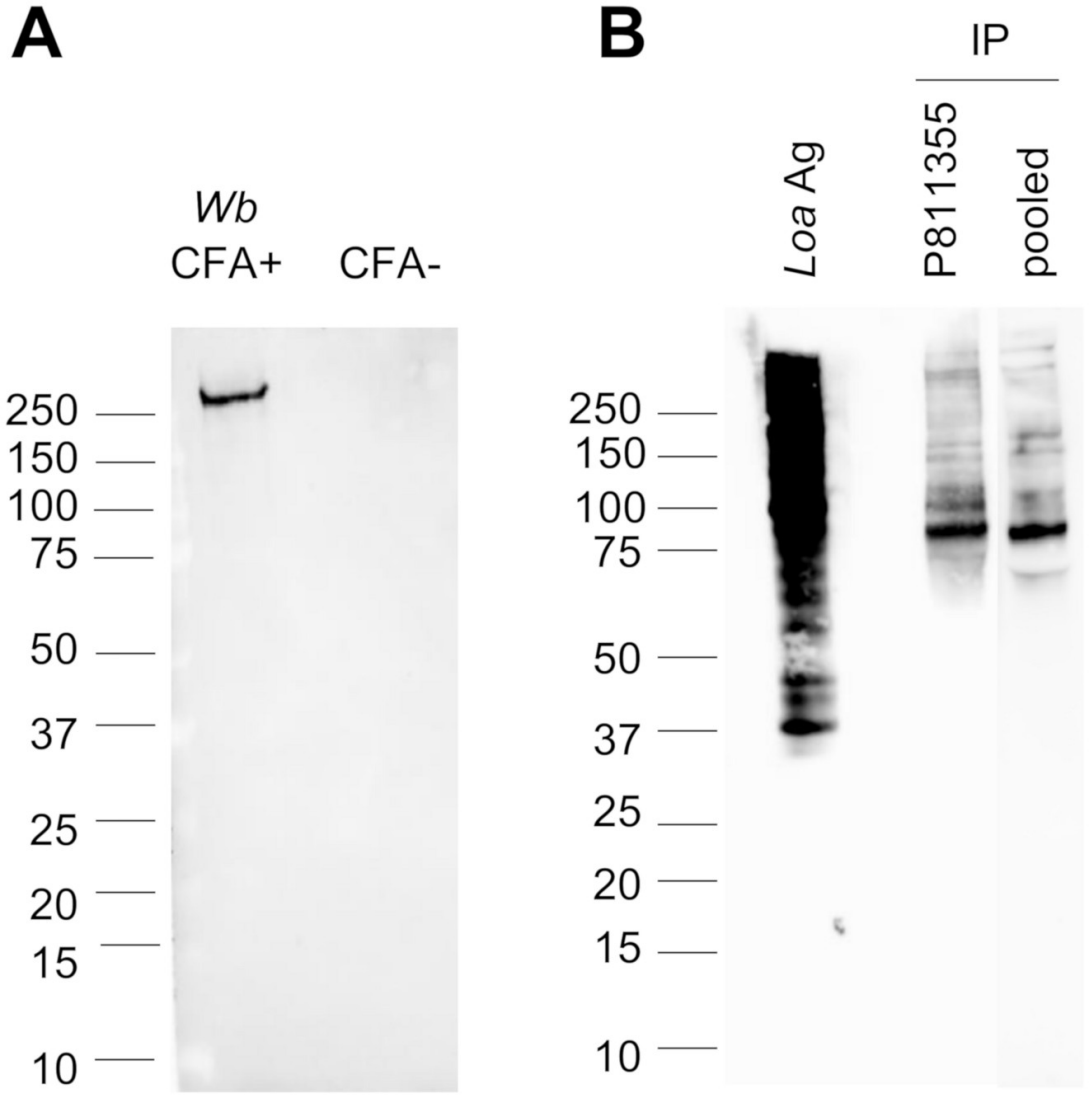
Loiasis sera positive by LF RDT contain multiple AD12 epitope-containing antigens. **(A)** AD12 western blot showing reactive bands in pooled sera from 13 individuals with *W*. *bancrofti* infection (*Wb* CFA+) or 12 uninfected individuals (CFA-). **(B)** Antigens from sample P811355 (355), and pooled cross-reactive sera. Soluble *L*. *loa* antigen (*Loa* Ag, 0.5 μg) served as a positive control. Because the antigen level was much higher in sample P811355 than in the pooled sample, different chemiluminescent exposure times were used for the same blot (45 seconds for soluble *L*. *loa* antigen and P811355; 15 minutes for the pooled serum sample).

We tested FTS-positive samples by western blot to visualize antigens reactive with AD12. Sample P811355, which had the highest antigen level, was analyzed separately; six other samples with weaker antigen signals by FTS were pooled (see Table 2) for analysis. We captured AD12 epitope-containing antigens by incubating these sera with agarose beads conjugated to DH6.5 monoclonal antibody, which recognizes the same carbohydrate epitope as AD12, and then detected the bound proteins by western blot using horseradish peroxidase-conjugated AD12 antibody. Both sample 811355 and the pooled serum sample contained many reactive proteins including a major band at ~80 kDa. This pattern is very different from that seen with sera from *W*. *bancrofti*-infected persons (Fig 3). Serum samples from LF RDT-negative loiasis serum, including five individuals from the Akonolinga/Awae field study who were previously *W*. *bancrofti* RDT positive but negative at the time of our study, were negative for AD12 containing antigens by western blot.

### Proteomic analysis of *L*. *loa* antigens immunoprecipitated from human serum with monoclonal antibody DH6.5

We next analyzed immunoaffinity-purified antigens from sample 811355 and the pooled sera by protease digestion and LC-MS/MS, matching MS spectra to both available *L*. *loa* genomes [17, 18]. After reconciling duplicated annotations between the two genomes, 220 *L*. *loa* proteins with two or more unique peptides were detected in the 811355 sample; ten were detected in the pooled sera. Seven proteins were detected in both samples. Table 3 lists the most abundant proteins identified; a list of all identified proteins is provided in S2 Table.

**Table 3:** Proteins identified in cross-reactive *L*. *loa* sera.

Unique peptides (total spectral counts)		Description	ID^1^	LOAG ID^2^	*C*. *elegans o*rtholog^3^
811355	Pooled				
76 (93)	2 (2)	Myosin heavy chain 4	EN70_1344	-	unc-54
29 (63)	21 (30)	Hypothetical zinc metalloprotease	EN70_10459	-	nas-14
21 (30)	2 (2)	Sugar transporter	EN70_10241	LOAG_09566	fgt-1
18 (21)	3 (3)	Tropomyosin	-	LOAG_17659	lev-11
2 (2)	1 (12)	Hypothetical zinc metalloprotease	EN70_10463^4^	-	nas-14
2 (19)	1 (12)	Hypothetical zinc metalloprotease	EN70_10462	-	nas-14
2 (17)	5 (5)	Myosin heavy chain	-	LOAG_18869	unc-54
41 (53)	-	Paramyosin	EN70_1595	LOAG_05509	unc-15
31 (34)	-	Mesocentin	-	LOAG_01560	dig-1
30 (40)	-	Chaperonin GroEL	EN70_9299	LOAG_17523	hsp-60
30 (34)	-	ATP synthase subunit beta	EN70_3459	-	atp-2
24 (24)	-	Spectrin protein 1	EN70_4597	LOAG_16411	add-1
22 (34)	-	Galectin-2	EN70_3647	LOAG_01794	lec-2
21 (28)	-	Heat shock protein 90	EN70_561	-	daf-21
21 (26)	-	ADP/ATP carrier protein	EN70_6911	LOAG_07239	ant-1.1
20 (20)	-	Spectrin beta chain	EN70_174	LOAG_04011	unc-70
19 (21)	-	Myosin light chain	EN70_10990	LOAG_04932	mlc-3
18 (26)	-	Myosin regulatory light chain 1	EN70_948	LOAG_03316	mlc-1
18 (20)	-	Calcium-transporting ATPase	EN70_10268	-	sca-1
17 (21)	-	Phosphate carrier protein	EN70_6829	LOAG_00237	F01G4.6
15 (17)	-	Sodium/potassium-transporting ATPase subunit alpha	EN70_7308	LOAG_07650	catp-4
14 (17)	-	ATP synthase subunit alpha	EN70_7528	LOAG_16780	H28O16.1
12 (13)	-	Hypothetical protein, adhesion domains	EN70_5928	-	dig-1
12 (12)	-	Chaperone DnaK	EN70_547	LOAG_16336	hsp-6
11 (14)	-	Elongation factor 1-alpha	EN70_10934	LOAG_08753	eef-1A.1
11 (13)	-	Disorganized muscle protein 1	EN70_901	LOAG_02014	dim-1
11 (13)	-	Enolase	EN70_8669	-	enol-1

^1^Bioproject PRNJA246086 (Talon et al 2014)

^2^Bioproject PRNJA60051 (Desjardin et al 2013)

^3^parasite.wormbase.org

^4^Nine peptides shared between EN70_10462 and EN70_10463

### Bioinformatic analysis

To further characterize the loiasis antigens captured by DH6.5 immuno-purification, we performed a gene ontology (GO) enrichment analysis using Blast2Go software [19, 25] with the published *L*. *loa* proteome as a comparator [17]. At least one GO term could be assigned for 206 of the 220 (94%) loiasis proteins identified. Table 4 shows the top ten GO terms enriched in each category. Compared to the whole *L*. *loa* proteome, our identified proteins were enriched for cytoplasmic, organelle, and cytoskeletal proteins. The over-represented molecular functions included structural and protein binding activity, as well as nucleoside enzymatic activity and purine nucleoside binding. The biological processes enriched in the IP were largely concerned with reproduction and early development.

**Table 4:** GO enrichment analysis of mass spectrometry hits.

GO ID	GO Name	Fold Change	p-value^1^
**Cellular Compartment**			
GO:0005737	cytoplasm	2.0	2.3E-17
GO:0044422	organelle part	2.2	8.1E-16
GO:0044446	intracellular organelle part	2.2	2.3E-14
GO:0044444	cytoplasmic part	2.1	6.9E-14
GO:0099512	supramolecular fiber	4.4	1.0E-11
GO:0099081	supramolecular polymer	4.4	1.2E-11
GO:0043228	non-membrane-bounded organelle	2.4	1.2E-11
GO:0043232	intracellular non-membrane-bounded organelle	2.4	1.2E-11
GO:0099080	supramolecular complex	4.3	1.4E-11
GO:0030016	Myofibril	7.4	6.3E-11
**Molecular Function**			
GO:0005198	structural molecule activity	3.9	1.8E-11
GO:0005515	protein binding	2.1	2.5E-08
GO:0017111	nucleoside-triphosphatase activity	2.8	2.9E-07
GO:0016462	pyrophosphatase activity	2.8	4.4E-07
GO:0016818	hydrolase activity, acting on acid anhydrides, in phosphorus-containing anhydrides	2.7	4.9E-07
GO:0003735	structural constituent of ribosome	4.0	5.1E-07
GO:0016817	hydrolase activity, acting on acid anhydrides	2.7	5.4E-07
GO:0035639	purine ribonucleoside triphosphate binding	1.9	2.5E-06
GO:0001883	purine nucleoside binding	1.9	2.6E-06
GO:0032550	purine ribonucleoside binding	1.9	2.6E-06
**Biological Processes**			
GO:0009792	embryo development ending in birth or egg hatching	3.5	1.4E-23
GO:0002119	nematode larval development	3.3	1.1E-21
GO:0002164	larval development	3.3	1.2E-21
GO:0009790	embryo development	3.2	1.9E-21
GO:0009791	post-embryonic development	3.3	3.1E-21
GO:0000003	reproduction	2.7	6.2E-20
GO:0048608	reproductive structure development	4.7	5.1E-19
GO:0061458	reproductive system development	4.7	5.1E-19
GO:0045137	development of primary sexual characteristics	4.6	2.5E-17
GO:0008406	gonad development	4.6	2.5E-17

^1^based on binomial distribution

Analysis with secretion signal prediction software SignalP, which searches for classical N-terminus secretion signals, and SecretomeP, which searches for non-classical secretion signals, predicted that only 69 of 220 (31%) of the loiasis proteins are likely to be secreted [22, 23]. In comparison, 246 of 500 (49%, 95% CI: 45%– 54%) randomly selected proteins from the *L*. *loa* genome are predicted to be secreted by these algorithms. Because SecretomeP and SignalP were designed to predict mammalian, not filarial, protein secretion, we also compared the proteins identified in our screen to the well-characterized *B*. *malayi* secretome [26–28]. One hundred five of the 220 *L*. *loa* proteins we identified have clear *B*. *malayi* homologs. Eighteen (17%) of these were present in the *B*. *malayi* secretome, twelve of which are reportedly secreted by Mf, but not by adult worms. Nearly all (92 of 105, 88%) of the *B*. *malayi* homologues show evidence of expression in the *B*. *malayi* proteome, and the 71 of 92 (77%), are expressed in all stages examined [29]. Taken together, these analyses suggest that most of proteins detected by our screen in loiasis sera are not secreted, and that their source may be either Mf or adult worms (or both).

Since our purification strategy involved capturing with a monoclonal antibody specific for the AD12 carbohydrate epitope, we examined the predicted glycosylation status of the *L*. *loa* proteins identified in human serum samples. Eighty-three percent were predicted by NetOGlyc 4.0 [20] to have O-linked glycosylation sites, compared to 77% (95% CI: 74%– 82%) of a random set of 500 *L*. *loa* proteins. NetNGlyc 1.0 software [21] predicted that 47% of the *L*. *loa* proteins we identified may have N-linked glycosylation, compared to 18% (95% CI: 14%–21%) of the randomly selected *L*. *loa* proteins. Thus, the majority of proteins identified in our screen were, as expected, predicted to be glycoproteins. The absence of predicted glycosylation sites on a minority (20/220, 9%) of the identified proteins suggests that a fraction of the proteins we identified are not decorated with the AD12 glycan epitope.

### Analysis of excretory-secretory products from cultured *L*. *loa* worms

**Fig 4:**
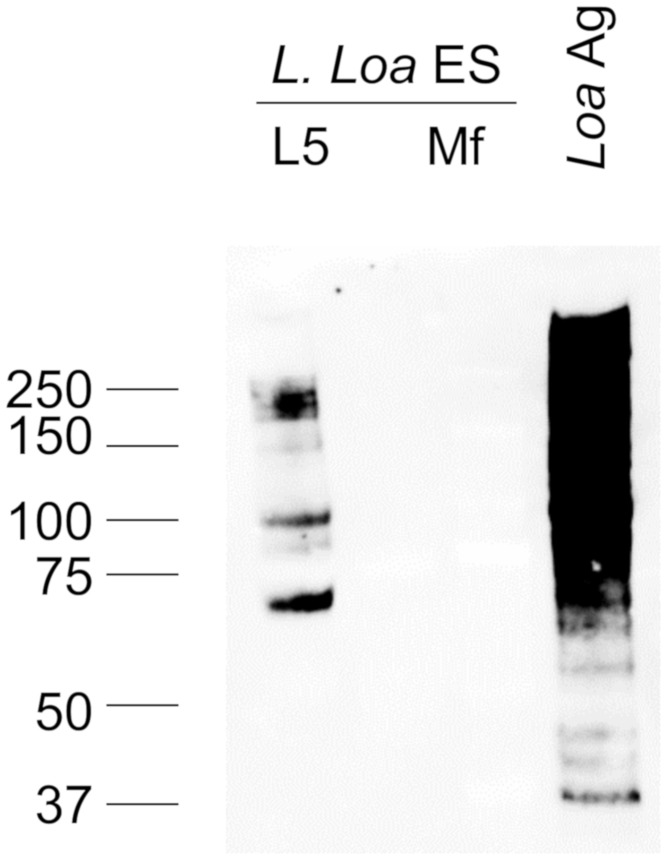
*L*. *loa* secretes glycoproteins reactive with monoclonal antibody AD12. Western blot of AD12 glycoproteins immunoaffinity purified from 1mL of culture supernatant (ES products) from *L*. *loa* adult worms (L5) or microfilaria (Mf). Soluble *L*. *loa* antigen (*Loa* Ag, 0.5 μg) served as a positive control.

Both *L*. *loa* Mf and explanted L5 (adult) worms cultured *ex vivo* secrete antigens that cross-react with *W*. *bancrofti* RDTs [15]. To identify these secreted antigens and to determine if they were among those present in patient sera, we examined the excretory/secretory (ES) products from adult female worms and from Mf cultured *in vitro*. Using DH6.5-conjugated beads to capture AD12 epitope-containing antigens, we detected several AD12-reactive antigens in the culture supernatants of adult female worms by western blot, but none were detected in Mf supernatants (Fig 4). LC MS/MS analysis identified four *L*. *loa* proteins in the adult (L5) supernatants that met the pre-specified requirement of having at least two unique peptides detected. Only one of these proteins, the *L*. *loa* ortholog of filarial antigen Av33, was also detected in loiasis sera (Table 5).

**Table 5:** Proteins identified in L5 stage *L*. *loa e*xcretory/secretory products.

Unique peptides (spectra)	Description	ID^1^	LOAG ID^2^	#peptides in loiasis serum^3^
5 (5)	Filarial antigen Av33-like	EN70_3801	LOAG_02368	5
2 (2)	U6 snRNA-associated Sm-like protein	EN70_10442	LOAG_05763	-
2 (2)	RAN GTP-binding nuclear protein	EN70_6080	-	-
2 (2)	14-3-3 zeta	EN70_4422	LOAG_05701	-

^1^Bioproject PRNJA246086 (Talon et al 2014)

^2^Bioproject PRNJA60051 (Desjardin et al 2013)

^3^S2 Table

## Discussion

Reactivity of circulating *L*. *loa* antigens with *W*. *bancrofti* RDTs is an impediment to successful LF elimination in loiasis-endemic areas in Central Africa, because a CFA prevalence of 1% or higher is considered to be evidence that the area is endemic for LF and should receive MDA [30]. In this study we sought to identify *L*. *loa* antigens that are responsible for false-positive results in LF RDTs. This work led to several unexpected observations. First, RDT cross-reactivity was absent in many persons previously ICT-positive despite persistence of high *L*. *loa* microfilarial loads. The mechanism of loss of cross-reactivity is unclear, but it does not appear to be due to masking of the AD12 epitope by antibodies. It also seems unlikely that changes in the performance characteristics of the ICT were responsible; we contacted Alere and were assured no changes in the manufacturing process or quality control testing of the ICT were made between 2013 and 2016. Second, unlike bancroftian filariasis, where RDT positivity is associated with a single circulating filarial glycoprotein, cross-reactive loiasis sera contain multiple AD12 epitope-containing *L*. *loa* antigens. Most of these antigens are not predicted to be secreted and are related to intracellular processes and structures. In addition, most are not specific to any particular stage of the filarial lifecycle. Only one of the *L*. *loa* antigens identified in cross-reactive sera was also detected in excretory/secretory products of *ex vivo* cultured *L*. *loa* adults.

These results suggest that the release of cross-reactive *L*. *loa* antigens occurs by a process or processes quite different from that of the *W*. *bancrofti* CFA [5]. We believe that the most likely mechanism for loiasis cross-reactivity with *W*. *bancrofti* RDTs is death of *L*. *loa* Mf and/or adult worms associated with release of multiple AD12 glycoproteins into circulation and that imbalance between release and clearance of these antigens accounts for their detection in the circulation of some loiasis patients. It is known that not all loiasis patients with active infection (as indicated by the presence of eye worms) have circulating *L*. *loa* Mf, and this is believed to be due to immune clearance of Mf. Perhaps immune-mediated clearance of Mf results in intermittent release of AD12 epitope-containing antigens. Another explanation would be that the antigens are released by the intermittent death of adult worms. Either of these mechanisms would be consistent with the observation that although there is a correlation between microfilarial load and RDT-positivity, not all persons with cross-reactive RDTs due to loiasis have detectable microfilaremia [7].

It is important to recognize that while the ICT and FTS rapid diagnostic tests detect a specific high molecular weight *W*. *bancrofti* glycoprotein in serum samples from persons infected with *W*. *bancrofti*, they do so by recognizing a carbohydrate epitope (the AD12 epitope) that is not unique to *W*. *bancrofti*. This epitope is present on multiple proteins in lysates of *Dirofilaria* and *Brugia* [5, 6]. While AD12 epitope-containing proteins are present in somatic antigen preparations and ES products of *L*. *loa* Mf and L3 larval stages [15], the developmental stage from which the cross-reactive antigens in serum originate remains unclear. Our set of 220 *L*. *loa* proteins resembles microfilarial or immature uterine expression signatures based on number of matches and total spectral counts from a stage specific study of *B*. *malayi* [29] and corresponds well with a *L*. *loa* Mf transcription analysis [18]. It seems likely that at least some of the proteins we detected are of microfilarial origin, which contrasts with Bancroftian filariasis in which the dominant circulating *W*. *bancrofti* antigen is produced primarily by adult worms [5, 31].

Several prior studies have reported circulating *L*. *loa* antigens in the blood [32–34] and urine [35] of persons with loiasis. Our study differed in that we sought to specifically capture the antigens responsible for *W*. *bancrofti* RDT cross-reactivity. It is not surprising, therefore, that our approach identified different circulating *L*. *loa* antigens compared to prior studies. For example, out of the 18 *L*. *loa* proteins identified by Drame et al. in urine from one *L*. *loa* microfilaremic individual, only two, peptidase M16 inactive domain-containing protein (LOAG_04876) and pyruvate kinase (LOAG_17249), were also found in our analysis [35].

Our study had some significant limitations. First, the difficulty of obtaining cross-reactive sera and the need to pool the samples that were not strongly positive has resulted in characterization of only two samples. Whether these findings are representative of all persons with *W*. *bancrofti* RDT cross-reactivity due to loiasis will require further study. Second, since all the sera we analyzed were from persons co-infected with *M*. *perstans*, it is impossible to rule out detection of some *Mansonella* proteins in our analyses. It seems unlikely, however, that cross-reactive *Mansonella* proteins were prominent, since *M*. *perstans* Mf counts were 2.5 to 92 times lower than *L*. *loa* Mf counts in the samples we analyzed (see Table 2). Furthermore, prior studies found no correlation between *M*. *perstans* microfilariemia and ICT or FTS positivity among patients with loiasis [7–9]. It would be interesting to see how many of the proteins identified in our MS analysis might also be part of the *M*. *perstans* proteome; unfortunately, there is no *M*. *perstans* genome available to address this issue at this time.

Second, we cannot be certain which of the antigens we identified actually contribute to RDT cross-reactivity. The immunoprecipitation conditions we used to capture cross-reactive antigen (4°C rocking overnight) differ from the RDTs (room temperature, 10 minutes). We chose overnight incubation in order to capture as many cross-reactive antigens as possible for downstream analysis. It is possible that shorter incubation times would capture fewer antigens. Additionally, because these antigens are glycoproteins, it is impossible to correlate the apparent MW, based on gel electrophoresis, with the proteins identified by shotgun mass spectrometry. Our data therefore identify *L*. *loa* antigens present in cross-reactive sera, but do not establish which antigens are necessary and/or sufficient to cause a cross-reactive RDT. The presence of a predominant ~80 kDa band in both the #811355 sera and the pooled sera suggests it may be a major contributor. Future studies with larger sample numbers will be necessary to address these questions.

One perplexing aspect of *W*. *bancrofti* RDT positivity in loiasis is that only a small percentage of samples from persons with loiasis cross-react. Our Akonolinga and Awae field study adds another piece to this puzzle, since it demonstrates that loiasis cross-reactivity may vary over time despite persistence of microfilaremia. As mentioned above, we feel the most plausible explanation for this is an imbalance between the intermittent release of *L*. *loa* antigens and the capacity of the host to clear these antigens. The number and varied intracellular locations and diverse functions of the antigens detected in cross-reactive sera suggest that the antigens are coming from dead or dying parasites and not being excreted or secreted from living worms.

In conclusion, this study has provided new information on cross-reactivity of CFA tests for LF in persons with loiasis. Prospective studies examining the duration of RDT cross-reactivity in persons with loiasis (and the factors associated with its development and resolution) may help to clear up unresolved issues. Our results also demonstrate a method for distinguishing between lymphatic filariasis and loiasis based on patterns of AD12-reactive antigens in human sera revealed by western blot. Additional research will be required to develop improved tests for the specific diagnosis of both of these infections.

## Supporting information

S1 Table Central Cameroon field study individual RDT results and Microfilaria counts. (XLSX)

S2 Table Complete list of *L*. *loa* proteins identified from cross-reactive loiasis serum. (XLSX)
